# Synthesis of a conjugated pyrrolopyridazinedione–benzodithiophene (PPD–BDT) copolymer and its application in organic and hybrid solar cells

**DOI:** 10.1007/s00706-017-1949-1

**Published:** 2017-03-30

**Authors:** Astrid-Caroline Knall, Andrew O. F. Jones, Birgit Kunert, Roland Resel, David Reishofer, Peter W. Zach, Mindaugas Kirkus, Iain McCulloch, Thomas Rath

**Affiliations:** 1 Institute for Chemistry and Technology of Materials (ICTM), NAWI Graz, Graz University of Technology, Stremayrgasse 9, 8010 Graz, Austria; 2 Department of Chemistry and Centre for Plastic Electronics, Imperial College London, Imperial College Road, London, SW7 2AZ UK; 3 Institute of Solid State Physics, Graz University of Technology, Petersgasse 16, 8010 Graz, Austria; 4 Institute of Analytical Chemistry and Food Chemistry, Graz University of Technology, Stremayrgasse 9, 8010 Graz, Austria; 5 King Abdullah University of Science and Technology (KAUST), SPERC, Thuwal, 23955-6900 Saudi Arabia

**Keywords:** Polymerization, Fullerenes, Materials science, Nanostructures, Photovoltaics, Hybrid nanomaterials

## Abstract

**Abstract:**

Herein, we describe the synthesis and characterization of a conjugated donor–acceptor copolymer consisting of a pyrrolopyridazinedione (PPD) acceptor unit, and a benzodithiophene (BDT) donor unit. The polymerization was done via a Stille cross-coupling polycondensation. The resulting PPD–BDT copolymer revealed an optical bandgap of 1.8 eV and good processability from chlorobenzene solutions. In an organic solar cell in combination with PC_70_BM, the polymer led to a power conversion efficiency of 4.5%. Moreover, the performance of the copolymer was evaluated in polymer/nanocrystal hybrid solar cells using non-toxic CuInS_2_ nanocrystals as inorganic phase, which were prepared from precursors directly in the polymer matrix without using additional capping ligands. The PPD–BDT/CuInS_2_ hybrid solar cells showed comparably high photovoltages and a power conversion efficiency of 2.2%.

**Graphical abstract:**

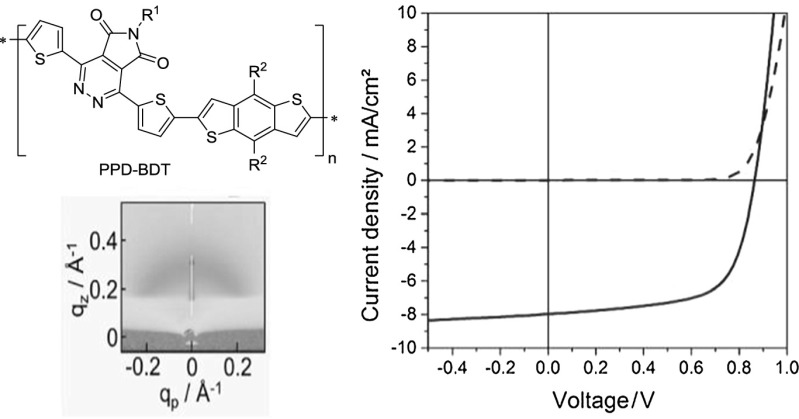

## Introduction

Tetrazines are highly electron-deficient acceptor units and several donor–acceptor (D–A) copolymers have been already successfully realized [1–7]. One current drawback, however, is their poor solubility, limiting the molecular weight achieved in polycondensation reactions and requiring electron-rich comonomers with large solubilising alkyl chains. Placing additional alkyl chains on the electron-deficient part of D–A copolymers enables alkyl chain engineering, which improves the morphology, solubility, and processability by varying the length of the alkyl chains. Therefore, we followed a similar approach as has been recently introduced for 4,7-bis(5-bromothiophen-2-yl)-2,1,3-benzothiadiazole, which also suffers from limited solubility. To solve this issue in the case of benzothiadiazole, 2,1,3-benzothiadiazole-5,6-dicarboxylic imide has been developed [8]. In this novel acceptor unit, solubilising alkyl chains of different lengths can be introduced and copolymers with this acceptor unit led to excellent efficiencies of over 8% without requiring processing additives or additional processing steps such as thermal annealing. In this study, we prepared pyrrolopyridazinedione (PPD) monomers via inverse Diels–Alder reaction (iEDDA) [9] of 3,6-bis(5-bromothiophen-2-yl)-1,2,4,5-tetrazine with N-alkylated maleimides. Typically, tetrazines act as electron-deficient dienes in iEDDA reactions which preferably react with strained, electron-rich dienophiles so that these reactions can be used for click chemistry due to their high reaction rates. Because the double bond in maleimide is strongly electron-deficient, a high amount of energy has to be put into the iEDDA reaction, i.e. the reaction has to be carried out at 160 °C in a high boiling solvent for several hours.

By introducing the additional imide unit, the electron-deficient character of the PPD unit is further enhanced compared to the parent tetrazine with the added possibility of introducing additional alkyl chains to improve and tune solubility as outlined above. Furthermore, due to the flanking thiophene units, conformation locks are avoided in the resulting copolymers.

Based on a recent report on tetrazine-based copolymers [2], benzo[1,2-*b*:4,5-*b′*]dithiophene (BDT) turned out to be a promising donor unit for the combination with the prepared PPD acceptor [10]. In recent years, the BDT unit emerged as a very interesting building block for the synthesis of conjugated copolymers [11]. BDT-based polymers turned out to be very efficient and power conversion efficiencies (PCEs) up to 11.2% could be already realized in organic solar cells [11, 12]. For a tetrazine-based BDT copolymer maximum PCEs of 5% could be obtained so far [2]. The planar and rigid structure of BDT makes it well suited for achieving high hole mobilities and this building block also allows tuning of molecular energy levels and optical band gaps of the polymers.

In this work, we investigate the performance of a PPD–BDT copolymer in organic solar cells in combination with PC_70_BM as well as in hybrid solar cells in combination with CuInS_2_ (CIS) nanocrystals. For the hybrid solar cells, the PPD–BDT/CIS absorber layer was prepared via an in situ route based on the thermal decomposition of precursors (copper and indium xanthates) directly in the conjugated polymer [13]. This direct route, in which no capping ligands for the nanocrystals are involved, leads to a nanocomposite layer, in which copper indium sulfide nanocrystals (3–5 nm in size) are well distributed and form a percolating network in the polymer matrix. Also partial agglomeration of the nanocrystals can be observed depending on the nanocrystal content in the layer [13–15] or depending on the alkyl moiety of the metal xanthate [16].

Using PSiF-DBT as conjugated polymer, PCEs of 2.8% could be obtained in combination with CIS nanocrystals [13]. In addition to conjugated polymers [17], also small molecular donors like p-DTS(FBTTh_2_)_2_ can be used as organic matrix [18] and the prepared solar cells exhibit good long-term stability [19]. Due to the low conversion temperature of the metal xanthates to the metal sulfide nanocrystals, in particular, if *n*-hexylamine is used as additive, also the preparation of flexible solar cells [15] and the fabrication of tandem solar cells [20] is possible using this in situ route.

## Results and discussion

1,4-Bis(5-bromothiophen-2-yl)-6-(2-decyltetradecyl)-5*H*-pyrrolo[3,4-*d*]pyridazine-5,7(6*H*)-dione (PPD, Scheme 1) was synthesized from 3,6-bis(5-bromothiophen-2-yl)-1,2,4,5-tetrazine (**1**) and *N*-(2-decyltetradecyl)-maleimide (**2**) in a similar fashion to Qun et al. using ortho-dichlorobenzene as solvent instead of diphenyl ether [21]. Due to the electron-deficient nature of **2**, a high reaction temperature had to be applied to facilitate the iEDDA reaction leading to the desired pyrrolopyridazine. The monomer could be purified by precipitation into chilled methanol and subsequent column chromatography using cyclohexane/ethyl acetate (20/1) as eluent.

**Figure:**
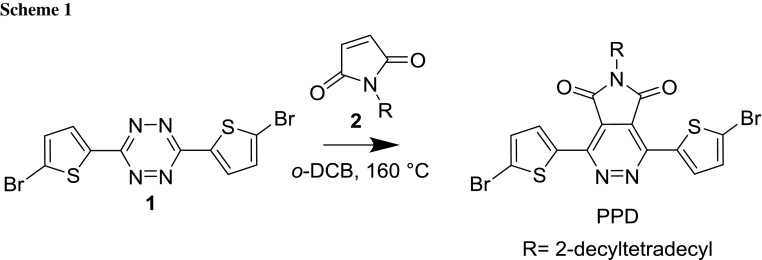

The PPD–BDT copolymer was synthesised using PPD and [4,8-bis[5-(2-ethylhexyl)thiophen-2-yl]benzo[1,2-*b*:4,5-*b*′]dithiophene-2,6-diyl]bis(trimethylstannane) (BDT, see Scheme 2) in a microwave-assisted Stille cross-coupling polycondensation, using tris(dibenzylideneacetone)dipalladium(0)/tris(*o*-tolyl)phosphine as catalyst and chlorobenzene as solvent, in 68% yield. Subsequently, the crude product was purified by Soxhlet extractions. The resulting polymer is well soluble in chloroform and chlorobenzene. The number average weight (*M*_n_) of the prepared PPD–BDT polymer is 24 kDa with a polydispersity index (PDI) of 1.9 (see Table 1).

**Figure:**
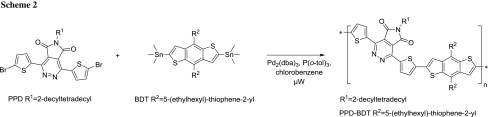

**Table 1:** Characteristic properties of the synthesized PPD–BDT copolymer

Yield/%	*M*_*n*_/kDa	PDI	Abs. max./nm	Abs. onset/nm	Bandgap/eV
68	24^a^	1.9	556	692	1.8

^a^Bimodal molecular weight distribution with maxima at 12 and 60 kDa

**Fig. 1:**
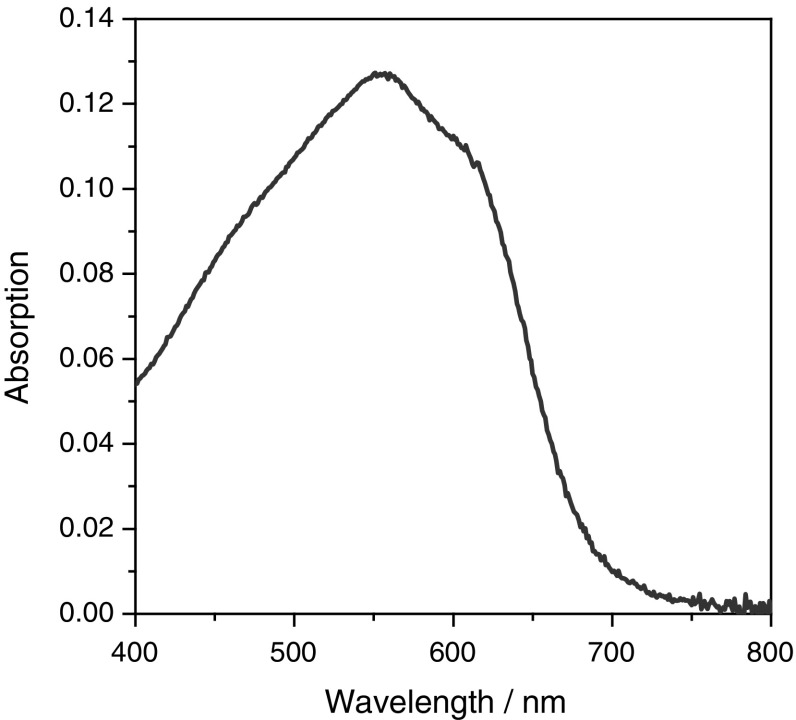
UV–Vis absorption spectrum of the prepared PPD–BDT copolymer

The UV–Vis absorption spectrum of PPD–BDT is shown in Fig. 1. The polymer has an absorption maximum at 567 nm and an absorption onset at about 700 nm which corresponds to an optical bandgap of 1.8 eV (see Table 1).

**Fig. 2:**
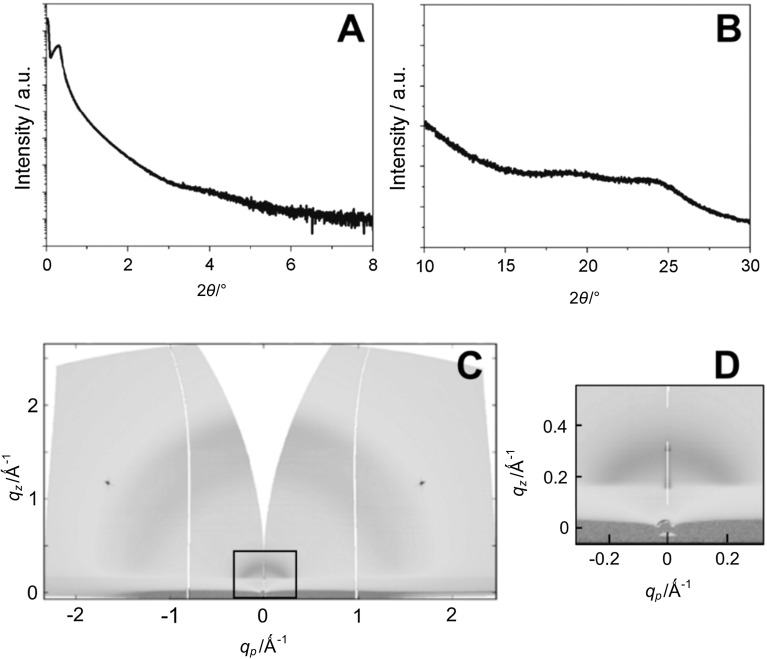
X-ray reflectivity data (**a**), specular X-ray diffraction data (**b**) and a 2D X-ray diffraction pattern (**c**, **d** zoom-in of area highlighted in **c**) of a drop-coated PPD–BDT film on a silicon wafer substrate

The crystallisation properties and molecular packing of the prepared conjugated polymer were investigated by X-ray reflectivity (XRR) and specular X-ray diffraction measurements on polymer thin films. Additionally, a 2D X-ray diffraction pattern was measured using synchrotron radiation at the XRD1 beamline at the Elettra synchrotron in Trieste, Italy, to study the structure in the in-plane direction. The XRR and specular X-ray diffraction measurements only give information about the packing in the out-of-plane direction (perpendicular to the substrate surface) and indicate a mostly amorphous nature of the polymer film (Fig. 2a, b), with no strong Bragg peaks visible. In the 2D diffraction pattern (Fig. 2c, d), no sharp in-plane peaks are observed. However, a weak ring-like feature is observed at *q* = 0.29 Å^−1^ with a sharper Bragg peak in the out-of-plane direction, which corresponds to a lattice spacing of 2.17 nm; the onset of the same peak can also be seen in the XRR data (Fig. 2a) at a 2*θ* value of ~4°. This indicates that there is some degree of preferred orientation of the molecules parallel to the substrate. Also, a broad ring-like feature of higher intensity at *q* ~ 1.6–1.7 Å^−1^, which shows differences in intensity along the ring and is more pronounced in the out-of-plane direction, is observed. This corresponds to a d-spacing of ~0.38 nm, which is indicative of π–π stacking and gives a hint for a preferred face-on orientation of the polymer chains. The sharp peaks at *q*_z_ = 1.2 Å^−1^ in Fig. 2c stem from the silicon substrate.

**Fig. 3:**
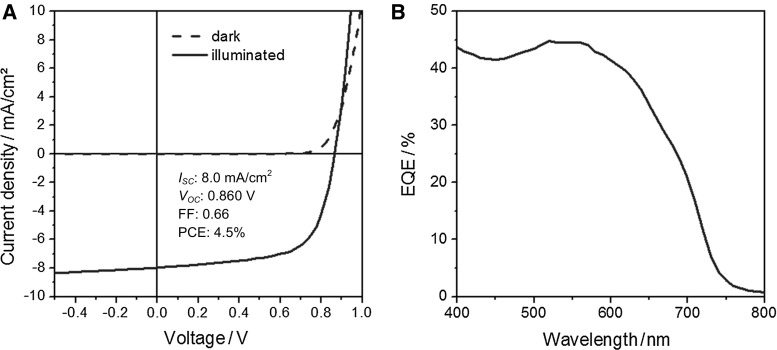
**a** IV-curves measured in the dark and under 100 mW/cm^2^ illumination and **b** EQE spectrum of a typical PPD–BDT/PC_70_BM solar cell

The performance of PPD–BDT in organic bulk heterojunction solar cells was investigated using PC_70_BM as acceptor and the solar cells were prepared in an inverted architecture (glass/ITO/ZnO/PPD–BDT—PC_70_BM/MoO_3_/Ag). The absorber layer was prepared from chlorobenzene solution containing 2% 1,8-diiodooctane (DIO), an additive, which influences the phase separation and morphology of the polymer/PCBM bulk heterojunction layer [22]. Due to the use of DIO, no annealing of the absorber layer was necessary and significantly higher short circuit currents (*I*_SC_) than without additive could be obtained. The prepared solar cells exhibit an *I*_SC_ of 8.0 mA/cm^2^, a photovoltage (*V*_OC_) of 860 mV and a fill factor (FF) of 0.66, which leads to a power conversion efficiency (PCE) of 4.5%. The EQE spectrum of the solar cell (Fig. 3b) shows an onset between 700 and 750 nm and exhibits a maximum at approx. 550–560 nm, which matches well with the absorption maximum of the PPD–BDT polymer (cf. Fig. 1).

The solar cell performance obtained with the prepared polymer in this work is higher than the PCE found by Zhang et al. with a polymer synthesized from the same building blocks in a recent study, where PCEs up to 3.66% were reached in a regular device architecture [10]. In the present study, we prepared the solar cells in an inverted device geometry and furthermore, the PPD–BDT obtained in our study had a higher molecular mass (*M*_n_ 24 kDa) compared to the previously published sample (*M*_n_ 11.9 kDa), which both might contribute to the increase in PCE.

As the variation of the alkyl chains on both electron-rich and electron-deficient components has a profound influence on blend morphology and consequently device performance [23], the performance of these solar cells can be potentially further increased by tuning the alkyl chain substitution pattern of the polymer. In particular, the introduction of shorter alkyl moieties (e.g. 2-ethylhexyl instead of 2-decyltetradecyl) into the PPD building block is envisaged.

**Fig. 4:**
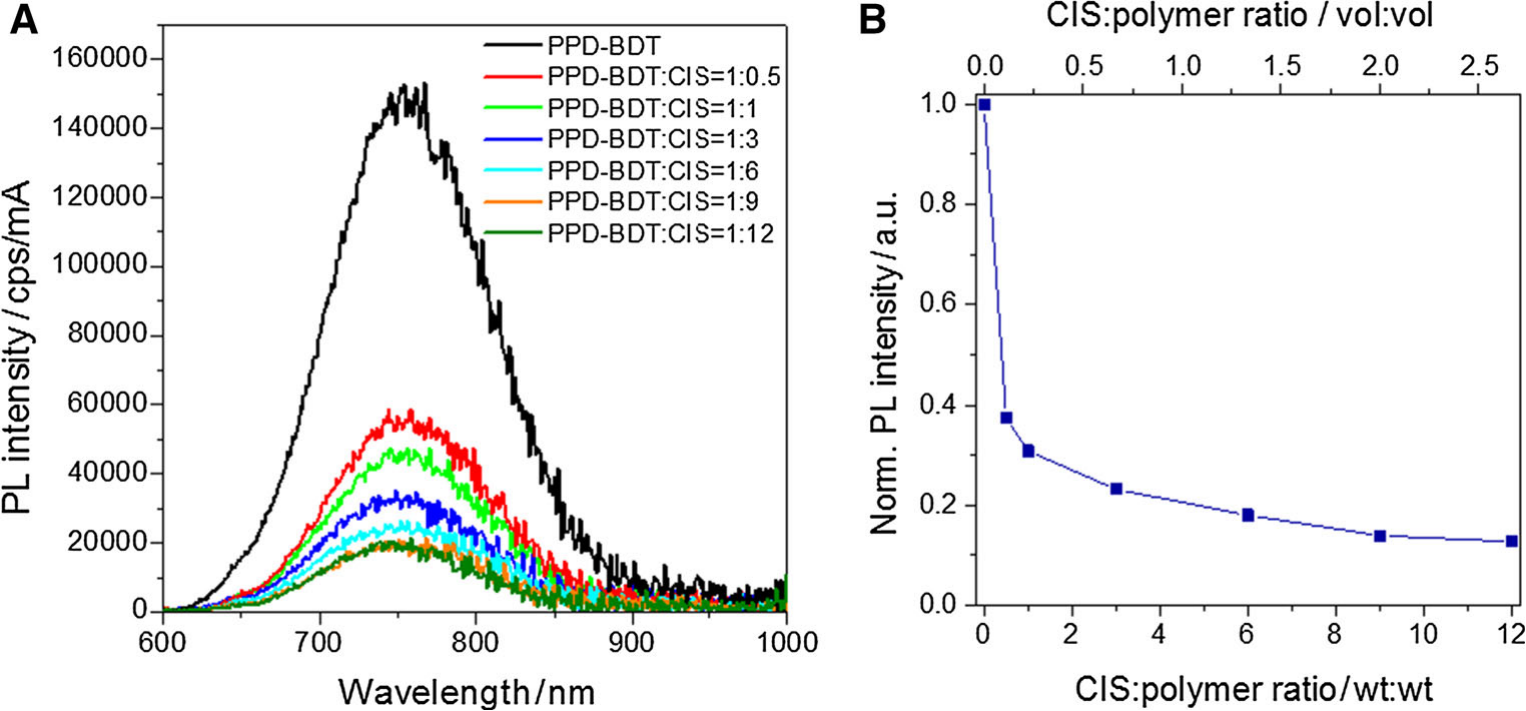
**a** Quenching of the PPD–BDT emission (excitation wavelength: 540 nm) in nanocomposite films with different polymer:CIS ratios and **b** the corresponding normalized integrated photoluminescence intensities plotted vs. the CIS:polymer ratio (wt:wt and vol:vol)

Furthermore, we evaluated the performance of the prepared PPD–BDT in hybrid solar cells in combination with CIS nanocrystals, which are promising inorganic non-fullerene acceptors due to the fact that all required elements are abundant and the nanoparticle synthesis can be straightforwardly performed using the in situ approach outlined in the introduction. To get an indication if charge transfer between the polymer and the nanocrystals occurs, a photoluminescence (PL) quenching study was performed. In Fig. 4a, b, it can be seen that in films with a PPD–BDT:CIS weight ratio of 1:0.5, the PL of the polymer is already quenched to about 37% of its initial value. For nanocomposite films with increased CIS nanocrystal content the PL is decreased to 14% where it remains more or less unchanged when going from a PPD–BDT:CIS ratio of 1:9 to 1:12 (wt:wt).

By comparing these values to previous studies, it is apparent that the PL quenching is not as efficient as when PSiF-DBT is used as conjugated polymer [13, 24], but more efficient than in PCDTBT/CIS layers [24]. A possible reason for the non-complete quenching of the photoluminescence of PPD–BDT by the CIS nanocrystals might be small domains in the nanocomposite film, in which the polymer is not well blended with the nanocrystals, similar to like it was already observed for PCDTBT [24].

**Fig. 5:**
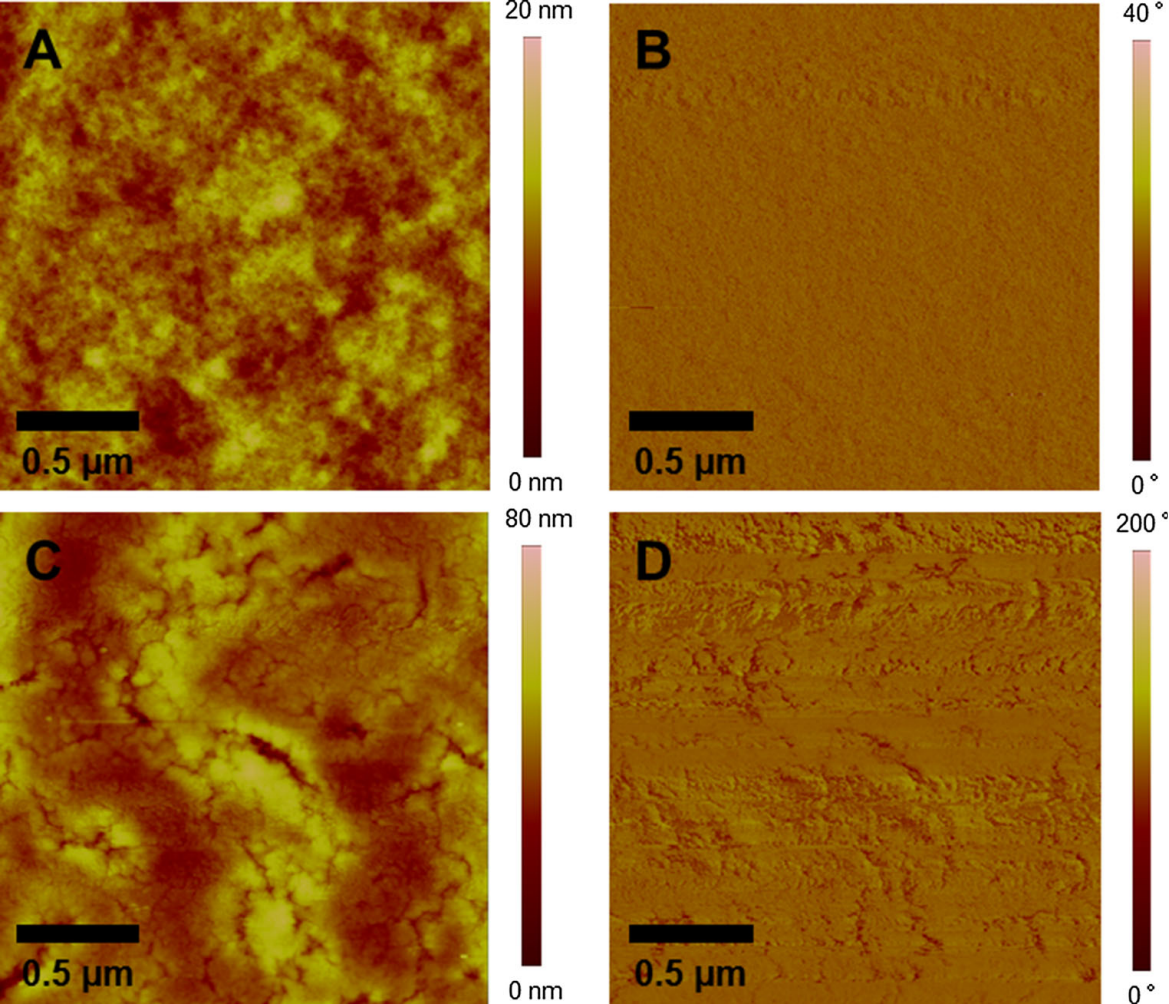
AFM images of PPD–BDT/PCBM (**a**, **b**) and PPD–BDT/CIS (**c**, **d**) films (**a**, **c** topography images; **b**, **d** phase images)

The AFM images in Fig. 5 also indicate that the phase separation in the PPD–BDT/CIS film is coarser than in the PPD–BDT/PC_70_BM film. Moreover, the surface of the hybrid film is rougher due to the conversion of the metal xanthates to the CIS nanocrystals directly in the film, which leads to a significant loss in volume of the layer because of the evaporation of volatile products of the metal xanthate decomposition out of the layer [13]. Some elongated depressions surrounded by elevated areas are found in the topography image and a similar pattern can be found in the phase image indicating domains with significantly higher polymer or nanocrystal concentration, respectively. In contrast to that, the phase image of the PPD–BDT/PC_70_BM film appears very homogeneous.

**Fig. 6:**
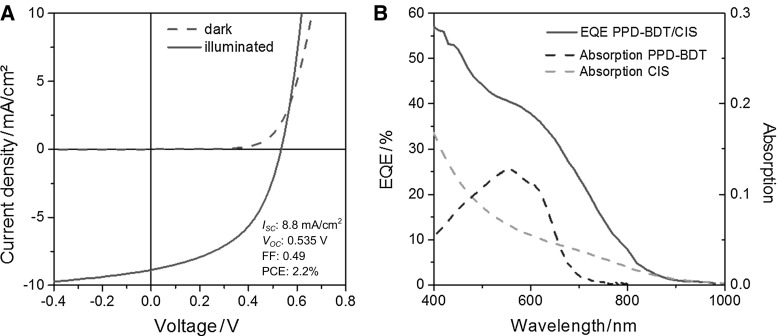
**a** IV-curves measured in the dark and under 100 mW/cm^2^ illumination of a typical PPD–BDT/CIS hybrid solar cell and **b** a corresponding EQE spectrum and absorption spectra of PPD–BDT and CIS nanocrystals

As the PL quenching experiments revealed that with a PPD–BDT:CIS ratio of 1:12 no further quenching of the PL can be obtained, we chose a weight ratio of 1:9 for the preparation of the hybrid solar cells, a weight ratio which already turned out to lead to good results for other polymers [13, 14]. The solar cells were prepared in the architecture glass/ITO/PEDOT:PSS/PPD–BDT—CIS/Ag and the IV curves of a typical PPD–BDT/CIS solar cell are shown in Fig. 6a. Interestingly, the obtained *I*_SC_ (8.8 mA/cm^2^) is slightly higher than observed for the organic solar cell (8.0 mA/cm^2^). This could stem from the additional absorption of the CIS nanocrystals in the absorber layer. By comparing the EQE spectra of the PPD–BDT/CIS and the PPD–BDT/PC_70_BM solar cells (Figs. 3b, 6b), it can be noticed that the onset in the EQE spectrum of the hybrid solar cells is already above 850 nm, while the onset in the organic solar cell is between 700 and 750 nm. The absorption maximum of the polymer at approx. 550–560 nm can be also recognized as a pronounced shoulder and the EQE spectrum keeps increasing towards lower wavelengths due to the additional absorption of the CIS nanocrystals (see Fig. 6b).

The overall PCE of the hybrid solar cell is, however, despite the higher *I*_SC_, significantly lower than for the organic solar cell, because of the lower *V*_OC_ and the lower FF and a value of 2.2% is obtained. However, it should be also pointed out that the *V*_OC_ of 535 mV is the highest observed so far for in situ prepared polymer/CIS hybrid solar cells in which stable Ag electrodes are used instead of Al electrodes, which lead to limited lifetimes of the devices [19].

## Conclusion

Strongly electron-deficient, thiophene-flanked 6-alkylpyrrolo[3,4-d]pyridazine-5,7-dione (PPD) was synthesized and successfully incorporated in alternating D–A copolymers in combination with a benzodithophene (BDT) donor using Stille cross-coupling polymerization. The observed high *V*_OC_ values and good fill factors in organic solar cells suggest that PPD monomers are valuable building blocks in D–A copolymers. Typically PCEs of 4.5% could be obtained with PPD–BDT/PC_70_BM absorber layers in organic solar cells. An approach to further optimize the efficiency of these solar cells is to improve the *I*_SC_. Therefore, polymers with shorter solubilizing alkyl chains are currently under investigation. Furthermore, also hybrid solar cells could be realized in combination with in situ prepared CIS nanocrystals. Also in this solar cell type, the prepared copolymer showed decent performance and good processability, which is a good incentive for further studies.

## Experimental

### Polymer synthesis

[4,8-Bis[5-(2-ethylhexyl)thiophen-2-yl]benzo[1,2-*b*:4,5-*b*′]dithiophene-2,6-diyl]bis(trimethylstannane) (BDT monomer) and 1,4-bis(5-bromothiophen-2-yl)-6-(2-decyltetradecyl)-5*H*-pyrrolo[3,4-*d*]pyridazine-5,7(6*H*)-dione (PPD monomer) were synthesized according to literature procedures [21, 25]. All other chemicals were purchased from commercial sources (Sigma Aldrich, VWR, Alfa Aesar) and used as received.

[4,8-Bis[5-(2-ethylhexyl)thiophen-2-yl]benzo[1,2-*b*:4,5-*b*′]dithiophene-2,6-diyl]bis(trimethylstannane) (183.7 mg, 0.2031 mmol), 164.1 mg of 1,4-bis(5-bromothiophen-2-yl)-6-(2-decyltetradecyl)-5*H*-pyrrolo[3,4-*d*]pyridazine-5,7(6*H*)-dione (0.2031 mmol), 4.42 mg Pd_2_(dba)_3_ (4.8 × 10^−3^ mmol), 6.3 mg (*o*-tol)_3_P (2.1 × 10^−2^ mmol), and 2 cm^3^ anhydrous chlorobenzene were placed inside a 5 cm^3^ glass tube and degassed for 20 min. This tube was then sealed and degassed with argon for another 10 min. The sealed tube was then placed in a Biotage Initator microwave reactor and subjected to the following temperature programme: 100 °C (2 min), 120 °C (2 min), 140 °C (5 min), 160 °C (5 min), and 180 °C (30 min). To end-cap the polymer chains, a few drops of bromobenzene were added (20 mm^3^) via syringe and the reaction mixture was heated sequentially at 100 °C (2 min), 120 °C (2 min), 140 °C (5 min), and 160 °C (5 min). Then, 30 mm^3^ phenyltrimethyltin was added and the reaction mixture was heated sequentially at 100 °C (2 min), 120 °C (2 min), 140 °C (5 min), and 160 °C (5 min).

The resulting dark purple solution was precipitated into methanol and the precipitated polymer was recovered by filtration directly into an extraction thimble. Soxhlet extractions were performed with acetone, hexanes, and chloroform. The majority of the polymer was dissolved in the chloroform fraction. This solution was concentrated to 2 cm^3^ and precipitated into methanol. This precipitation was repeated twice.

GPC (chlorobenzene, 80 °C): *M*_n_ = 24,000 g/mol, *M*_w_ = 46,000 g/mol; yield 169 mg (68%).

### Solar cell preparation

PPD–BDT/PC_70_BM solar cells were prepared on glass/ITO substrates (Xinyan Technology Ltd., Hong Kong), which were cleaned in isopropanol in an ultrasonic bath followed by plasma etching (Femto, Diener Electronics). Next, a planar ZnO film was prepared on the glass/ITO substrates by spin coating a precursor solution (1 g zinc acetate, 0.28 g ethanolamine, 10 cm^3^ 2-methoxyethanol) followed by a temperature treatment of the films (200 °C, 45 min) in ambient atmosphere [26]. Afterwards, the PPD–BDT/PC_70_BM films were prepared by spin coating (5 mg/cm^3^ PPD–BDT and 5 mg/cm^3^ PC_70_BM dissolved in chlorobenzene, additive: 1,8-diiodooctane, 2%). No subsequent temperature treatment was necessary. The solar cell preparation was finished by thermal evaporation of a MoO_3_ (10 nm)/Ag (100 nm) layer at a base pressure of 8 × 10^−6^ mbar.

The PPD–BDT/CIS hybrid solar cells were fabricated in the device architecture glass/ITO/PEDOT:PSS/PPD–BDT—CIS/Ag. Therefore, glass/ITO substrates with a sheet resistance of 10 Ω/sq were used, which were cleaned in deionized water and isopropanol in an ultrasonic bath followed by O_2_ plasma cleaning. The PEDOT:PSS layer (Clevios P VP.Al 4083, Heraeus) was spin coated on the glass/ITO layer in ambient atmosphere and subsequently annealed at 150 °C for 10 min in a glove box. In a next step, the hybrid films were prepared by spin coating of a chlorobenzene solution containing PPD–BDT, copper and indium xanthate (concentration of PPD–BDT in the precursor solution: 5 mg/cm^3^; the weight ratio of PPD–BDT:CuInS_2_ was 1:9 considering a full conversion of the xanthates to CuInS_2_ nanocrystals) and subsequent thermal treatment (temperature program: 15 min heating from room temperature to 195 °C followed by 15 min at 195 °C) on a programmable heating plate (MCS 66, CAT Ingenieurbüro M. Zipperer GmbH). The used copper xanthate (copper *O*-(2,2-dimethylpentan-3-yl)dithiocarbonate) and indium xanthate (indium *O*-(2,2-dimethylpentan-3-yl)dithiocarbonate) have been purchased from Aglycon KG, where they were synthesized according to a previously published procedure [13]. In the last preparation step, silver electrodes (100 nm) were deposited via thermal evaporation at a base pressure of 8 × 10^−6^ mbar.

### Characterization techniques

The UV–Vis absorption spectrum of the conjugated polymer was measured with a Shimadzu UV-1800 UV spectrophotometer. The number-average molecular weight (*M*_n_) was determined with an Agilent Technologies 1200 series GPC in chlorobenzene at 80 °C using two PL mixed B columns in series. The system was calibrated against narrow weight-average dispersity (*D*_*W*_ < 1.10) polystyrene standards.

The X-ray reflectivity and specular X-ray diffraction measurements were done on a PANalytical Empyrean system using Cu K_α_ radiation (*λ* = 0.154 nm) with a beam height of 100 μm. On the primary side, a multilayer X-ray mirror was used to generate a parallel beam. On the secondary side, an anti-scatter slit and 0.02 rad Soller slit were used with a PANalytical PIXcel^3D^ detector. 2D X-ray diffraction patterns were obtained at the XRD1 beamline at the ELETTRA synchrotron in Trieste, Italy [27]. X-rays with a wavelength of *λ* = 1.0 Å were used with an incident angle of *α*_*i*_ = 2°; a Pilatus 2 M detector was used to record scattered intensities.

Photoluminescence spectra were measured in ambient atmosphere on a FluoroLog 3 spectrofluorometer from Horiba Scientific equipped with a NIR-sensitive R2658 photomultiplier from Hamamatsu (300–1050 nm). The PL spectra were corrected for the spectral sensitivity of the detector.

IV curves of the prepared solar cells were recorded in inert atmosphere using a Keithley 2400 SourceMeter, a custom made Lab-View software and a Dedolight DLH400D lamp with a spectrum very similar to AM1.5G (light intensity: 100 mW/cm^2^, determined using a KippZonen-CMP-11 pyranometer, no spectral mismatch was considered). EQE spectra were recorded using a MuLTImode4 monochromator (AMKO) equipped with a Xenon lamp and a Keithley 2400 source meter.

Atomic force microscopy (AFM) was performed on a Veeco Multimode Quadrax MM AFM (Bruker; Billerica, MA, USA) in tapping mode using NCH-VS1-W silicon cantilevers (force constant: 42 N/m; resonance frequency: 296.3 kHz) from NanoWorld AG, Neuchatel, Switzerland.
